# A population‐level application of a method for estimating the timing of HIV acquisition among migrants to Australia

**DOI:** 10.1002/jia2.26127

**Published:** 2023-06-14

**Authors:** Jonathan M. King, Kathy Petoumenos, Timothy Dobbins, Rebecca J. Guy, Richard T. Gray, Steven J. Nigro, Damin Si, Byron Minas, Skye McGregor

**Affiliations:** ^1^ The Kirby Institute UNSW Sydney Sydney New South Wales Australia; ^2^ School of Population Health UNSW Sydney Sydney New South Wales Australia; ^3^ Communicable Diseases Branch Health Protection NSW Sydney New South Wales Australia; ^4^ Communicable Diseases Branch Prevention Division Queensland Health Brisbane Queensland Australia; ^5^ Communicable Disease Control Directorate, Department of Health WA Perth Western Australia Australia

**Keywords:** HIV epidemiology, public health, HIV prevention, sexually transmitted infections/diseases, men who have sex with men, key and vulnerable populations

## Abstract

**Introduction:**

Australia has set the goal for the virtual elimination of HIV transmission by the end of 2022, yet accurate information is lacking on the level of HIV transmission occurring among residents. We developed a method for estimating the timing of HIV acquisition among migrants, relative to their arrival in Australia. We then applied this method to surveillance data from the Australian National HIV Registry with the aim of ascertaining the level of HIV transmission among migrants to Australia occurring before and after migration, and to inform appropriate local public health interventions.

**Methods:**

We developed an algorithm incorporating CD4^+^ T‐cell decline back‐projection and enhanced variables (clinical presentation, past HIV testing history and clinician estimate of the place of HIV acquisition) and compared it to a standard algorithm which uses CD4^+^ T‐cell back‐projection only. We applied both algorithms to all new HIV diagnoses among migrants to estimate whether HIV infection occurred before or after arrival in Australia.

**Results:**

Between 1 January 2016 and 31 December 2020, 1909 migrants were newly diagnosed with HIV in Australia, 85% were men, and the median age was 33 years. Using the enhanced algorithm, 932 (49%) were estimated to have acquired HIV after arrival in Australia, 629 (33%) before arrival (from overseas), 250 (13%) close to arrival and 98 (5%) were unable to be classified. Using the standard algorithm, 622 (33%) were estimated to have acquired HIV in Australia, 472 (25%) before arrival, 321 (17%) close to arrival and 494 (26%) were unable to be classified.

**Conclusions:**

Using our algorithm, close to half of migrants diagnosed with HIV were estimated to have acquired HIV after arrival in Australia, highlighting the need for tailored culturally appropriate testing and prevention programmes to limit HIV transmission and achieve elimination targets. Our method reduced the proportion of HIV cases unable to be classified and can be adopted in other countries with similar HIV surveillance protocols, to inform epidemiology and elimination efforts.

## 1 INTRODUCTION

UNAIDS has set the ambitious target to eliminate the global transmission of HIV by 2030 [1] and Australia is aiming for “virtual elimination” by the end of 2022 [2]. Encouragingly, the number of HIV notifications in Australia has declined between 2016 and 2019, largely attributed to a decline in the number of notifications among Australian‐born gay men and other men who have sex with men, due to the successful scale‐up of HIV treatment as prevention programmes, and more recently HIV pre‐exposure prophylaxis [3]. By contrast, there was no decline in the number of people newly diagnosed with HIV among people born overseas (hereafter referred to as migrants), including migrant gay men and other men who have sex with men, and heterosexuals [3]. Similar findings have been reported in other countries [4].

In response, there has been a strong focus on further understanding the HIV epidemiology among migrants. Data on country of birth and language spoken at home are becoming increasingly complete variables (>90% in 2020) in Australian HIV surveillance data, allowing for detailed analyses stratified by country of birth, and informing the development of culturally appropriate public health programmes. However, accurate information on whether HIV was acquired before or after arrival in Australia is still lacking. These data are vital, as for transmissions which occur prior to arrival, the focus should be on strategies to increase timely testing and diagnosis, such as HIV self‐testing [5]. For HIV transmissions which occur after arrival, the focus should be more on prevention, such as HIV pre‐exposure prophylaxis.

In Australia, three variables may provide some information about the timing of acquisition relative to migration. The first is a clinical presentation at diagnosis, such as symptoms consistent with HIV seroconversion illness, suggesting the infection must have been acquired in recent months. The second is the past HIV testing history of the person diagnosed, which provides a window for when HIV acquisition occurred. The third variable is the clinician's estimate for the place of HIV acquisition, which is based on assumptions, and may be subject to interviewer and/or recall bias [6].

Another commonly used method to estimate the timing of HIV acquisition relative to the year of migration is CD4^+^ T‐cell decline back‐projection modelling, which can be applied to HIV surveillance data [6, 7, 8, 9]. This method uses the CD4^+^ T‐cell count taken close to the time of a person's HIV diagnosis to back project the likely time a person acquired HIV, and then based on the year of arrival, classifies HIV acquisition occurring before or after migration. At a population level, this method has been used in a range of countries [6, 7, 8, 9, 10, 11, 12, 13, 14, 15]. However, there are some limitations when used alone, including the reliance on complete CD4^+^ T‐cell counts in surveillance data. Also, CD4^+^ T‐cell counts can decline considerably around the time of seroconversion [16], which could falsely indicate a person acquired HIV many years ago, rather than recently. We aimed to develop a new algorithm which used a CD4^+^ T‐cell decline back‐projection model, combined with data on clinical presentation, past HIV testing history and clinician estimate of the place of HIV acquisition. We then applied the algorithm to newly diagnosed HIV cases among migrants to Australia, to ascertain the level of transmission occurring before and after migration, and to inform appropriate local public health interventions.

## 2 METHODS

### 2.1 Study population

The study population included all people newly diagnosed with HIV who had their diagnosis notified (hereafter referred to as notifications) recorded in the Australian National HIV Registry between 1 January 2016 and 31 December 2020, with a reported country of birth as outside Australia and aged over 15 years at the time of diagnosis. We chose 2016 as the earliest year because the migrant year of arrival has been collected by most Australian states and territories since 2016 (South Australia being the only jurisdiction not collecting the year of arrival). Notifications with an HIV exposure of vertical transmission were excluded because for these people, the acquisition likely occurred around the time of birth. Socio‐demographic and clinical data were obtained from the National HIV Registry, including age, gender, country of birth, year of arrival in Australia, state/territory of diagnosis, exposure classification, date of diagnosis, CD4^+^ T‐cell count, date of CD4^+^ T‐cell count, previous HIV testing history (date of most recent HIV test with a negative result), clinical status at the time of diagnosis (including seroconversion illness), date of onset of primary HIV (indicated by clinical symptoms) and the clinician estimate for country of HIV acquisition for migrants diagnosed with HIV.

### 2.2 Classifying time since HIV acquisition relative to migration

**Figure 1:**
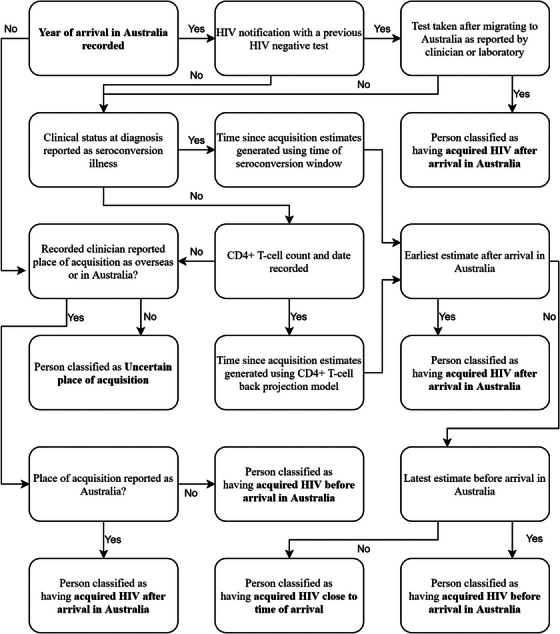
Algorithm for classifying time of HIV acquisition.

The time since HIV acquisition was estimated using the following algorithm (see Figure 1). For those with a record of a clinician or laboratory‐reported negative HIV test since arrival in Australia, their acquisition was assumed to have occurred after arrival in Australia. For those without an HIV test since arrival in Australia, earliest and latest estimates for the time since HIV acquisition were generated, using either the CD4^+^ T‐cell count close to the time of diagnosis or the clinical status at HIV diagnosis.

**Figure 2:**
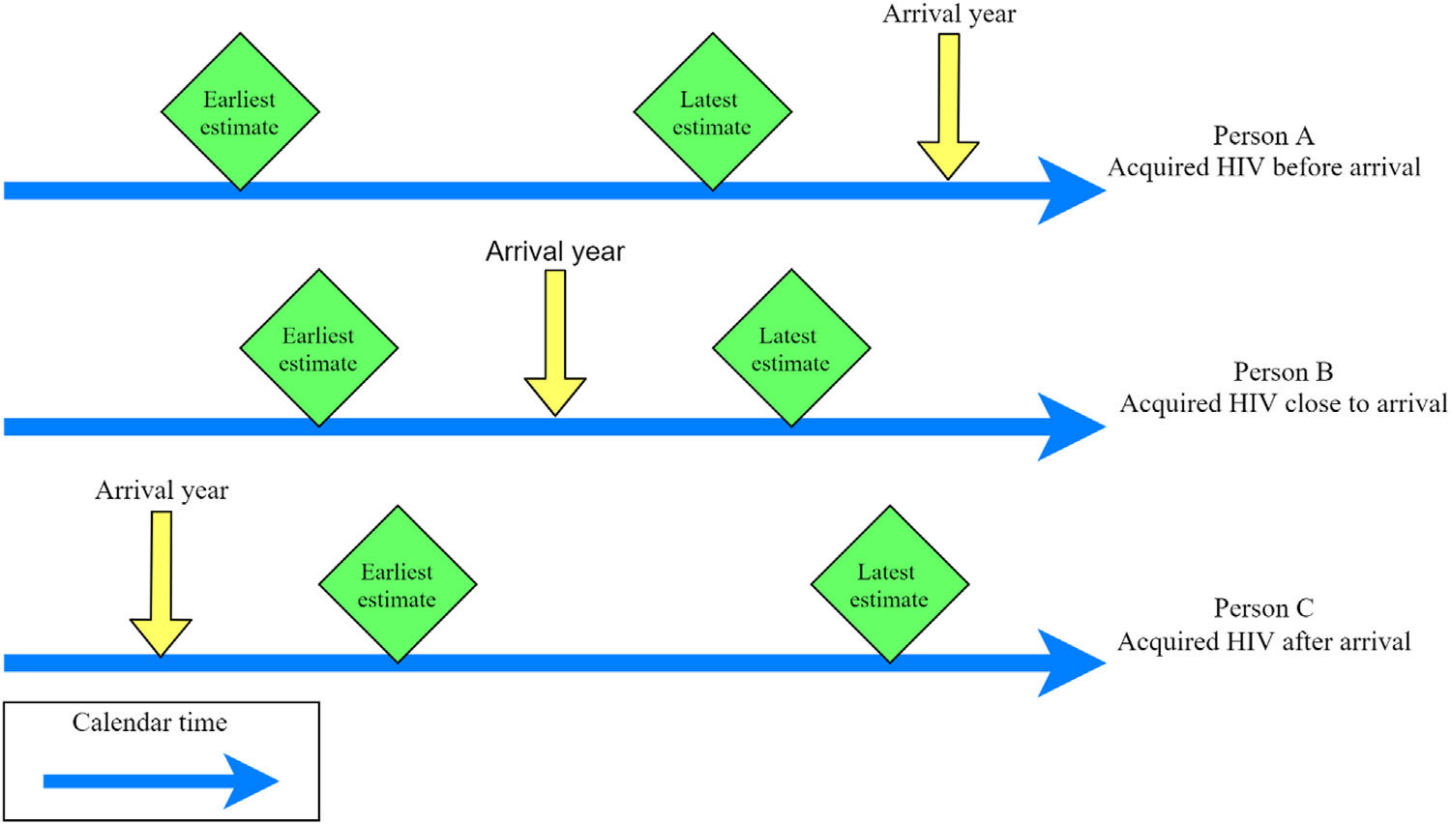
Time of acquisition estimates relative to the date of migration.

These estimates were then compared with the mid‐point for the reported year of arrival, to determine whether HIV was acquired pre‐ or post‐arrival in Australia (Figure 2). If the latest estimate for the time since HIV acquisition was before the year of arrival, that person was classified as having acquired HIV before arrival. If the earliest estimate for the time since HIV acquisition was after the year of arrival that person was classified as having acquired HIV after arrival in Australia. For those whose year of arrival was between the earliest and latest estimate of time since HIV acquisition, their time of acquisition relative to the date of migration was designated close to arrival in Australia (acquired HIV within 6 months either side of arrival).

For individuals presenting with seroconversion illness (diagnosed according to clinical symptoms in combination with a positive HIV test), an indication of primary HIV at the time of diagnosis, the latest estimate of time since HIV acquisition was assumed to be 14 days because the minimum time to produce a positive result for a fourth‐generation HIV test post‐acquisition is 2 weeks [17]. The mean time from acquisition to primary HIV has been estimated to be around 25.8 days with the mean duration of acute HIV estimated to be around 16.8 days [18]. These times were combined to make the earliest estimate of time since the acquisition of 43 days.

For individuals not presenting with seroconversion illness or a negative HIV test after migration, but with a reported CD4^+^ T‐cell count taken within 3 months of diagnosis, a back‐projection model using a CD4^+^ T‐cell rate of decline was used to estimate the time since acquisition [7]. This back‐projection model was developed using European HIV surveillance data, where at least two CD4^+^ T‐cell counts were taken prior to the initiation of antiretroviral therapy and estimates the rate of CD4^+^ T‐cell decline because of progressing HIV infection. To account for CD4^+^ T‐cell fluctuations around the time of seroconversion, CD4^+^ T‐cell counts were only included in the development of the model if they were taken at least 3 months post‐seroconversion [7]. Further, in developing the model, CD4^+^ T‐cell counts were only included if participants had recorded a negative HIV test within 1 year of recording a positive HIV test. The resulting model estimates the earliest and latest times since seroconversion (with 1 year between the two estimates), accounting for age (*S_a_*) and region of birth (*S_r_*) by adjusting the rates of CD4^+^ T‐cell decline (*S_a_*). The back‐projection model equations to estimate the earliest and latest time since acquisition are given below:

$$\mathrm{Earliest}\;\mathrm{time}\;=\frac{\sqrt{U}-\sqrt{first\;CD4\;cell\;count}}{S_{b}+S_{r}+S_{a}\left(age\;at\;diagnosis\right)}+1.25$$

$$\mathrm{Latest}\;\mathrm{time}\;=\frac{\sqrt{L}-\sqrt{first\;CD4\;cell\;count}}{S_{b}+S_{r}+S_{a}\left(age\;at\;diagnosis\right)}+0.25$$

For notifications missing data that would otherwise enable classification for the timing of HIV acquisition, the clinician estimate for country of acquisition was used as a proxy, where available (Figure 1).

### 2.3 Statistical analysis

The effect of augmenting the CD4^+^ T‐cell back‐projection model output with (1) the testing and clinical data, and then (2) the testing and clinical data with the clinician estimate for the place of HIV acquisition (the enhanced algorithm) was examined using descriptive statistics. Using the enhanced algorithm output, we summarized the socio‐demographic characteristics of migrants, stratified by the estimated time of HIV acquisition, relative to the date of migration.

To validate the use of the clinician estimate as a proxy for the algorithm when the algorithm using testing history and clinical data classified the notification as uncertain, a benchmark against Cohen's kappa was used to assess concordance between the two measures [19]. This concordance analysis was conducted where the algorithm classification and the clinician estimate were uncertain and missing, respectively. All data analysis was conducted using Stata version 16 [20].

### 2.4 Ethical considerations

This study was reviewed and approved by the University of New South Wales Human Research Ethics Committee. The study data were sourced from the Australian National HIV Registry and were collected as part of routine HIV surveillance. These data were de‐identified prior to use in our study and consent was not sought from those notified with HIV.

## 3 RESULTS

### 3.1 Study population

Of the 4341 notification records in the National HIV Registry (excluding 13 notifications with an exposure of vertical transmission) aged 15 years and older at the time of diagnosis, and with a diagnosis date between 1 January 2016 and 31 December 2020, 1909 (44%) were recorded as migrants (Table 1). Of these 1909 notifications, the median age at HIV diagnosis was 33 years (interquartile range: 28−42), 85% were recorded as male, 14% as female and 1% as transgender. By region of birth, 34% were born in Southeast Asia, 17% in South, Central or North Asia, 13% in Europe, 13% in Africa or the Middle East, 11% in South or Central America, 10% in the Pacific and 1% in North America. Just over two‐thirds of notifications (69%) had a reported exposure classification of male‐to‐male sexual contact, around a quarter (26%) were attributed to heterosexual contact, with the remainder attributed to injecting drug use (1%) or other/missing exposure classification (4%). Between 2016 and 2019, the number of migrant HIV notifications fluctuated between 376 and 428 per year. However, in 2020, there were 291 HIV notifications among migrants (Table 1).

**Table 1:** Characteristics of migrants notified with HIV by estimated time of acquisition

	Characteristic	Acquired HIV after arrival in Australia (row %)	Acquired HIV before arrival in Australia (row %)	HIV acquisition close to arrival (row %)	Uncertain time of acquisition (row %)	Total
Total		932 (49%)	629 (33%)	250 (13%)	98 (5%)	1909
Age group	15−19	5 (26%)	9 (47%)	3 (16%)	2 (11%)	19
	20–29	233 (38%)	228 (37%)	118 (19%)	33 (5%)	612
	30–39	305 (44%)	258 (38%)	95 (14%)	29 (4%)	687
	40–49	192 (62%)	80 (26%)	24 (8%)	15 (5%)	311
	50+	197 (70%)	54 (19%)	10 (4%)	19 (7%)	280
Gender	Men	828 (51%)	499 (31%)	215 (13%)	73 (5%)	1615
	Women	89 (33%)	125 (46%)	32 (12%)	25 (9%)	271
	Transgender	15 (65%)	5 (22%)	3 (13%)	0 (0%)	23
Years since immigration^a^	Less than 2 years	90 (16%)	360 (62%)	117 (20%)	12 (2%)	579
	2−4 years	111 (34%)	110 (34%)	94 (29%)	11 (3%)	326
	5−9 years	134 (67%)	34 (17%)	29 (15%)	3 (2%)	200
	10–19 years	193 (91%)	7 (3%)	10 (5%)	3 (1%)	213
	20 years plus	267 (97%)	4 (1%)	0 (0%)	3 (1%)	274
Age at migration	0−14 years	177 (97%)	3 (2%)	1 (1%)	2 (1%)	183
	15–24 years	235 (55%)	99 (23%)	83 (19%)	11 (3%)	428
	25–34 years	265 (38%)	290 (42%)	127 (18%)	15 (2%)	697
	35 years plus	118 (42%)	123 (43%)	39 (14%)	4 (1%)	284
Exposure classification	Male‐to‐male sex	692 (62%)	391 (35%)	186 (17%)	40 (4%)	1123
	Heterosexual contact	196 (45%)	205 (47%)	61 (14%)	34 (8%)	435
	Injecting drug use	16 (76%)	4 (19%)	0 (0%)	1 (5%)	21
	Other	28 (35%)	29 (36%)	3 (4%)	23 (29%)	80
Region of birth	Africa	117 (47%)	91 (37%)	23 (9%)	16 (6%)	247
	Americas (South/Central)	80 (40%)	80 (40%)	37 (18%)	5 (2%)	202
	Americas (North)	13 (62%)	2 (10%)	5 (24%)	1 (5%)	21
	Asia (Southeast)	261 (40%)	267 (41%)	87 (13%)	42 (6%)	657
	Asia (Other)	144 (44%)	109 (33%)	58 (18%)	17 (5%)	328
	Europe	179 (70%)	40 (16%)	24 (9%)	11 (4%)	254
	Pacific	138 (69%)	40 (20%)	16 (8%)	6 (3%)	200
CD4^+^ T‐cell count at diagnosis(cells/μl)^b^	0−199	154 (34%)	268 (59%)	23 (5%)	12 (3%)	457
	200−349	153 (33%)	268 (59%)	24 (5%)	12 (3%)	457
	350−499	160 (40%)	186 (47%)	46 (12%)	8 (2%)	400
	500 or greater	188 (49%)	80 (21%)	111 (29%)	3 (1%)	382
State/territory	NSW/ACT	359 (50%)	226 (32%)	119 (17%)	12 (2%)	716
	NT	13 (43%)	14 (47%)	3 (10%)	0 (0%)	30
	QLD	177 (59%)	83 (28%)	39 (13%)	2 (1%)	301
	SA	35 (47%)	34 (46%)	0 (0%)	5 (7%)	74
	TAS/VIC	262 (43%)	209 (34%)	60 (10%)	75 (12%)	606
	WA	86 (47%)	63 (35%)	29 (16%)	4 (2%)	182
Year of diagnosis	2016	227 (53%)	118 (28%)	59 (14%)	21 (5%)	425
	2017	180 (48%)	134 (36%)	45 (12%)	17 (5%)	376
	2018	176 (45%)	130 (33%)	61 (16%)	22 (6%)	389
	2019	192 (45%)	155 (36%)	55 (13%)	26 (6%)	428
	2020	157 (54%)	92 (32%)	30 (10%)	12 (4%)	291

^a^ Not including 317 notifications missing year of arrival in Australia.

^b^ Not including 247 notifications missing CD4^+^ T‐cell count within 90 days of initial diagnosis.

### 3.2 Comparing estimation methods

Of the 1909 migrant notifications, 1592 (83%) had a recorded year of arrival in Australia, 1662 (87%) had a recorded CD4^+^ T‐cell count taken within 3 months of first diagnosis, 167 (9%) had a clinical record of HIV seroconversion and 221 (12%) had a record of a negative HIV laboratory test after arrival in Australia. Using the CD4 T‐cell back‐projection model alone, 622 (33%) notifications were estimated to have acquired HIV after arrival in Australia, 472 (25%) were estimated to have acquired HIV before arrival in Australia, while 321 (17%) were estimated to have acquired HIV close to the time of arrival (Table 2). Meanwhile, 494 (26%) were classified with an uncertain time of acquisition due to missing data, including 247 (13%) missing CD4 T‐cell count data and/or 317 (17%) missing year of arrival.

**Table 2:** Estimation methods for the time of HIV acquisition among migrants

	Clinician estimate	CD4 T‐cell back‐projection model (%)	CD4 T‐cell back‐projection model with clinical data^a^ (%)	Enhanced algorithm^b^ (%)
HIV acquired after arrival in Australia	831 (44%)	622 (33%)	732 (38%)	932 (49%)
HIV acquired before arrival in Australia	820 (43%)	472 (25%)	452 (24%)	629 (33%)
HIV acquired close to the time of arrival	−	321 (17%)	250 (13%)	250 (13%)
Missing/Uncertain	258 (14%)	494 (26%)	475 (25%)	98 (5%)
Total	1909 (100%)	1909 (100%)	1909 (100%)	1909 (100%)

^a^ CD4 T‐cell back‐projection model combined with HIV testing history and clinical presentation.

^b^ CD4 T‐cell back‐projection model combined with HIV testing history, clinical presentation and clinician estimate.

By augmenting the CD4 T‐cell back‐projection model with clinical data (testing history and clinical status presentation) into an algorithm, the proportion classified as having acquired HIV after arrival increased from 33% to 38%, and the proportion classified as acquired HIV close to arrival decreased from 17% to 13%. There was little change in the proportions classified as acquired HIV before arrival or uncertain (Table 2). By further enhancing the algorithm with the clinician‐reported place of acquisition, the proportions classified as acquiring HIV after arrival in Australia, and before arrival in Australia, increased from 38% to 49% and 24% to 33%, respectively. Meanwhile, the proportion classified as uncertain reduced from 25% to 5% (Table 2).

### 3.3 Timing of HIV acquisition relative to migration, using the enhanced algorithm

Using the enhanced algorithm incorporating testing history, clinical data (clinical presentation, CD4^+^ T‐cell count taken close to the time of diagnosis and clinician estimate for the place of HIV acquisition), 932 migrants (49%) were estimated to have acquired HIV after their arrival in Australia, 629 (33%) were estimated to have acquired HIV before arrival in Australia and 250 (13%) migrants were estimated to have acquired HIV close to their arrival in Australia. Another 98 migrants (5%) were unable to be classified in either category due to missing data (Table 1).

By gender, a higher proportion of transgender migrants diagnosed with HIV were estimated to have acquired HIV after arrival in Australia (65%, *n* = 15) compared with men (51%, *n* = 828) and women (33%, *n* = 89). By age group, a higher proportion of migrants aged 40−49 years and 50 years and older were estimated to have acquired HIV after arrival in Australia (62% and 70%, respectively), compared with younger age groups (below 45% for those aged 39 years or younger). By region of birth, a higher proportion of those born in Europe, the Pacific and North America were estimated to have acquired HIV after arrival in Australia (70%, 69% and 62%, respectively) compared with those born in South or Central America and Southeast Asia (40%, each). By exposure classification, a higher proportion of notifications with HIV acquisition attributed to injecting drug use and male‐to‐male sex were estimated to have acquired HIV after arrival (76% and 53%, respectively), compared with notifications attributed to heterosexual sex (40%).

### 3.4 Algorithm and the clinician estimate concordance

When excluding notifications with either an algorithm classification of uncertain time of acquisition (*n* = 475) or missing a clinician estimate for the place of acquisition (*n* = 258), *fair agreement* was shown between the algorithm and the clinician estimates for place of acquisition (*n* = 1064, Percent agreement: 71%; Expected agreement: 50%; κ = 0.42 95% CI [0.37, 0.47]) (see Table S1).

## 4 DISCUSSION

### 4.1 Principal results

We used CD4^+^ T‐cell decline back‐projection model, augmented with clinical status, HIV testing history, and clinician estimate of the country of HIV acquisition to estimate the timing of HIV acquisition among migrants to Australia newly diagnosed with HIV. Using this enhanced algorithm, 49% of migrants acquired HIV after arrival, 33% acquired HIV before arrival, 13% of migrants acquired HIV close to their arrival, and for 5% of migrants, the time of acquisition was uncertain. One‐fifth (20%) of HIV notifications otherwise unable to be classified with a place of HIV acquisition were able to be classified by using the clinician estimate for the place of HIV acquisition. Focusing on the 48% who acquired HIV after arrival, this compares to 32% using the CD4^+^ T‐cell decline back‐projection model alone, and 38% using the CD4^+^ T‐cell decline back‐projection model in combination with clinical status, and HIV testing history.

Other studies estimating the time of HIV acquisition have used a CD4^+^ T‐cell decline back‐projection model [6, 7, 8, 11] with others also incorporating viral load data [9, 15], testing history [10] and behavioural data [13, 14]. However, this is the first study that we are aware of that combines such a model with testing history, clinical presentation at the time of diagnosis and the clinician estimate for the place of HIV acquisition sourced from routine surveillance data to estimate the time since HIV acquisition.

Previous estimates for the proportion of migrants diagnosed with HIV acquiring HIV after arrival, obtained using CD4^+^ T‐cell decline back‐projection models incorporating European surveillance data, range widely from 18% to 63%, a range which our estimate of 49% falls within [6, 7, 8, 9, 10, 13, 14]. As reported in previous studies, post‐arrival HIV acquisition was more common among those who were older at diagnosis compared with those who were younger. Similarly, post‐arrival acquisition was more common among those reporting male‐to‐male sexual exposure compared with those reporting heterosexual exposure [6, 7, 13].

Variations in estimates presented previously and presented here may reflect differences in HIV epidemic profiles, years for data collection and regional migration patterns (and, therefore, ethnic diversity). Migration patterns over the study period were also influenced by COVID‐19 pandemic‐related impacts, including changes to sexual behaviours, closed international borders and changes in testing patterns [21]. Notably, between 2019 and 2020, the number of migrants diagnosed with HIV decreased by 30%, and for this reason, the interpretation of trends relating to HIV acquisition by year was not addressed.

With almost half of the migrants (49%) newly diagnosed with HIV estimated to have acquired HIV after arriving in Australia, our findings clearly indicate the importance of sustained efforts to prevent HIV acquisition among migration populations. These efforts may include culturally appropriate programmes to increase access to HIV pre‐exposure prophylaxis, ensure timely testing and treatment for those living with HIV, and reducing stigma. We also found a third of migrants (33%) were estimated to have acquired HIV before arrival in Australia, while another 13% acquired HIV close to their arrival in Australia. Many migrants arriving in Australia face significant challenges in accessing healthcare and are unaware of HIV testing options, especially migrants from high HIV prevalence countries and non‐English speaking backgrounds [22, 23]. For migrants who are already living with HIV but remain undiagnosed when they arrive in Australia, the focus should be on strategies to support regular HIV testing, including HIV self‐testing, which has been shown to be an acceptable model in this subgroup [24].

### 4.2 Strengths

Our enhanced algorithm has a number of strengths. First, using past HIV testing history, and a record of HIV seroconversion, resulted in a more accurate classification compared to the CD4^+^ T‐cell back‐projection model alone. Further, for those presenting with primary HIV at diagnosis, the uncertainty around CD4^+^ T‐cell count declines associated with primary HIV rather than late diagnoses was removed. Second, by augmenting the CD4^+^ T‐cell back‐projection model with clinical data readily available in the Australian National HIV Registry, we were not solely reliant on the availability of CD4^+^ T‐cell count, and other variables required by the CD4^+^ T‐cell back‐projection model. Previous studies using similar methods have only included HIV cases in their study population that contain complete CD4 T‐cell count and year of arrival data [6, 8]. Such methods might lead to systematic biases if those with missing data are more likely to belong to specific subgroups. Using our enhanced algorithm, 5% of migrant notifications were classified as having uncertain timing of the acquisition, compared to 26% in the CD4^+^ T‐cell back‐projection model. Also, knowing the number, and proportion of cases is important for service provision planning.

### 4.3 Limitations

There are also some limitations to consider when interpreting our findings. The clinical estimate of the likely place of acquisition may have been provided by the person diagnosed many years after they arrived in Australia, and thus subject to recall bias. Also, assumptions may have been made by the diagnosing clinician, resulting in interviewer bias. However, when the algorithm is otherwise unable to determine the place of acquisition by more objective measures, the clinician estimate is a reasonable proxy and has been used elsewhere [25]. It should be acknowledged that the diagnosis of primary HIV relied on clinical symptoms and case history rather than laboratory evidence meaning that some cases may have been misdiagnosed and hence misclassified. Future population‐wide estimates for the place of acquisition may be made using molecular sequences from HIV drug resistance assays [26].

Another potential limitation may relate to individual variations in CD4^+^ T‐cell counts at diagnosis due to ethnic background. The CD4^+^ T‐cell count back‐projection model was developed using European HIV surveillance data and factored in the ethnic background of those notified. Because ethnic background can affect both CD4^+^ T‐cell counts and rates of CD4^+^ T‐cell decline [27], slightly differing estimates may have been generated if the CD4^+^ T‐cell back‐projection model was developed using Australian surveillance data.

Compared to other methods to estimate the place of acquisition, the CD4^+^ T‐cell count back‐projection used in our algorithm model uses fewer covariates [13, 14]. These other methods have the advantage of using detailed data obtained from cohort and behavioural studies and may produce more accurate estimates of the time since HIV acquisition. However, similar to methods used in a recently published study [9], our algorithm uses routinely collected national HIV surveillance data and can produce estimates to routinely report against key epidemiological indicators [28]. Lastly, in the development of the CD4^+^ T‐cell projection model, only participants with at least two CD4^+^ T‐cell counts were included meaning that bias may have been introduced by excluding those with fewer CD4^+^ T‐cell counts.

## 5 CONCLUSIONS

Our results show that almost half of the migrants diagnosed with HIV between 2016 and 2020 were estimated to have acquired HIV after arrival in Australia, showing that Australia still has a long way to go to achieve virtual elimination of HIV transmission, that is local HIV transmission. Further, a third of cases were estimated to have acquired HIV before arriving in Australia meaning that to reduce transmission, newly arrived migrants must have access to acceptable HIV testing options to engage in care as soon as practicably possible. Our method can also be easily used and adapted for routine surveillance reporting in Australia and elsewhere. With a more accurate method now available to estimate the timing of HIV acquisition, new national indicators should be developed based on the algorithm output.

## COMPETING INTERESTS

The authors declare no competing interests.

## AUTHORS’ CONTRIBUTIONS

JMK, SM and RJG conceived the idea for the study. JMK contributed to the data curation and writing the original draft. JMK and RTG contributed to the formal analysis. SM, KP and TD contributed to supervision. All authors contributed to the design of the methodology, review and editing.

## Supporting information


**Table S1**:Algorithm model and clinician estimate for time/place of acquisition excluding those classified as missing or uncertain.

## Data Availability

The data used in our study are personal health data and, therefore, are not shared.
