# Supplementary material for: A population‐level application of a method for estimating the timing of HIV acquisition among migrants to Australia
Source: J Int AIDS Soc. 2023 Jun 14;26(6):e26127. doi: 10.1002/jia2.26127 (PMC10267571; doi:10.1002/jia2.26127)
Supplement: Supplementary file 1 — Table S1:Algorithm model and clinician estimate for time/place of acquisition excluding those classified as missing or uncertain. [file JIA2-26-e26127-s001.docx]

Supplementary Table 1 Algorithm model and clinician estimate for time/place of acquisition excluding those classified as missing or uncertain^†^.

|  | Clinician estimate for place of acquisition | | |
| --- | --- | --- | --- |
| Algorithm estimate for time of acquisition^‡^ | Australia | Outside Australia | Total |
| After arrival in Australia | 448 | 224 | 663 |
| Before arrival in Australia | 84 | 308 | 399 |
| Total | 532 | 532 | 1064 |

^†^Excluding notifications classified as uncertain place of acquisition by the algorithm or missing by the clinician estimate; ^‡^Includes the CD4+ back-projection model, testing history and clinical presentation
